# Mechanical ventilation-induced alterations of intracellular surfactant pool and blood–gas barrier in healthy and pre-injured lungs

**DOI:** 10.1007/s00418-020-01938-x

**Published:** 2020-11-13

**Authors:** Jeanne-Marie Krischer, Karolin Albert, Alexander Pfaffenroth, Elena Lopez-Rodriguez, Clemens Ruppert, Bradford J. Smith, Lars Knudsen

**Affiliations:** 1 Institute of Functional and Applied Anatomy, Hannover Medical School, Carl-Neuberg Str. 1, 30625 Hannover, Germany; 2 Biomedical Research in Obstructive and Endstage Lung Disease Hannover (BREATH), Member of the German Center for Lung Research, Hannover, Germany; 3 Institute for Vegetative Anatomy, Charite, Berlin, Germany; 4 Department of Internal Medicine, Justus-Liebig University, Giessen, Germany; 5 Universities of Giessen and Marburg Lung Center (UGMLC), German Centre for Lung Research (DZL), Giessen, Germany; 6 Department of Bioengineering, University of Colorado Denver, Anschutz Medical Campus, Aurora, USA; 7 Department of Pediatric Pulmonary and Sleep Medicine, School of Medicine, University of Colorado, Aurora, USA

**Keywords:** Mechanical ventilation, Alveolar epithelial type II cells, Lamellar bodies, Surfactant, Blood–gas barrier, Stereology

## Abstract

Mechanical ventilation triggers the manifestation of lung injury and pre-injured lungs are more susceptible. Ventilation-induced abnormalities of alveolar surfactant are involved in injury progression. The effects of mechanical ventilation on the surfactant system might be different in healthy compared to pre-injured lungs. In the present study, we investigated the effects of different positive end-expiratory pressure (PEEP) ventilations on the structure of the blood–gas barrier, the ultrastructure of alveolar epithelial type II (AE2) cells and the intracellular surfactant pool (= lamellar bodies, LB). Rats were randomized into bleomycin-pre-injured or healthy control groups. One day later, rats were either not ventilated, or ventilated with PEEP = 1 or 5 cmH_2_O and a tidal volume of 10 ml/kg bodyweight for 3 h. Left lungs were subjected to design-based stereology, right lungs to measurements of surfactant proteins (SP−) B and C expression. In pre-injured lungs without ventilation, the expression of SP-C was reduced by bleomycin; while, there were fewer and larger LB compared to healthy lungs. PEEP = 1 cmH_2_O ventilation of bleomycin-injured lungs was linked with the thickest blood–gas barrier due to increased septal interstitial volumes. In healthy lungs, increasing PEEP levels reduced mean AE2 cell size and volume of LB per AE2 cell; while in pre-injured lungs, volumes of AE2 cells and LB per cell remained stable across PEEPs. Instead, in pre-injured lungs, increasing PEEP levels increased the number and decreased the mean size of LB. In conclusion, mechanical ventilation-induced alterations in LB ultrastructure differ between healthy and pre-injured lungs. PEEP = 1 cmH_2_O but not PEEP = 5 cmH_2_O ventilation aggravated septal interstitial abnormalities after bleomycin challenge.

## Introduction

The pulmonary surfactant system plays a critical role in reducing end-expiratory surface tension at the alveolar air–liquid interface as well as in minimizing respiratory workload during inspiration (Bachofen and Schürch 2001). These physiological functions are essential for alveolar micromechanics, defined as the morphological changes of alveoli during the respiratory cycle (Matuszak et al. 2020). The surface tension-lowering properties of surfactant stabilize alveolar dimensions even at low lung volumes so that the gas exchange surface area remains stable. The ventilation distribution across alveoli is homogenized, preventing stress concentrations and minimizing mechanical stresses acting on the very thin and fragile blood–gas barrier (Knudsen and Ochs 2018; Mead et al. 1970; Albert et al. 2019). Surfactant is a complex mixture consisting of 90% lipids, mainly phospholipids, and 10% proteins (Lopez-Rodriguez and Pérez-Gil 2014). Among the protein fraction, the hydrophilic surfactant proteins B (SP-B) and C (SP-C) which are critical for the biophysical properties of surfactant, are essential for normal pulmonary structure, function, and alveolar micromechanics (Rühl et al. 2019; Ruwisch et al. 2020). In general, the intra-alveolar surfactant pool responsible for biophysical and immunological functions can be differentiated from the intra-cellular surfactant pool within the alveolar epithelial type II (AE2) cells (Ochs 2010). Pulmonary surfactant, including both lipids and surfactant proteins, is synthesized, stored and secreted by AE2 cells (Perez-Gil and Weaver 2010; Ochs et al. 2020). AE2 cells possess a unique organelle for storing surfactant lipids and hydrophobic surfactant proteins called lamellar bodies (LB) which are the ultrastructural correlate of the intracellular surfactant pool. Lamellar bodies are modified lysosomes, ensconced in a limiting membrane and filled with onion-like and tightly packed biomembranes comprised of the surfactant lipids including the hydrophobic surfactant proteins. Lipid transporters such as the glycoprotein Abca3 (ATP-binding cassette class 3) located within the limiting membrane are essential for the biogenesis of LB. Mutations in the *Abca3* gene have been shown to result in ultrastructurally abnormal LB which become smaller and more numerous (Beers et al. 2017).

The intra-alveolar surfactant pool is regulated very tightly. Upon a stimulus, e.g., mechanical stretch due to a deep sigh, the limiting membrane of the LB fuses with the apical plasma membrane of the AE2 cell and releases its content into a thin liquid layer of an averaged thickness of approximately 140 nm, the so-called hypophase, into the alveolar space (Bastacky et al. 1995; Ochs et al. 2020; Dietl and Haller 2005). Within the alveolar space, the concentrically packed biomembranes unwrap to form a three-dimensional lattice-like structure termed tubular myelin. Tubular myelin represents a surfactant reservoir within the hypophase from which the lipid layer at the air–liquid interface originates. Used intra-alveolar surfactant is mainly recycled by AE2 cells and, to a lesser degree, degraded by alveolar macrophages as well as AE2 cells. Based on the electron microscopic criteria, dense multivesicular bodies can be differentiated from electron-lucent multivesicular bodies (Williams 1984). In AE2 cells, dense multivesicular bodies have been linked with degradation pathways of surfactant; while, electron-lucent multivesicular bodies are suggested to have a sorting function during recycling but might also be part of the de novo synthesis pathway of surfactant (Rooney et al. 1994; Weaver et al. 2002). In essence, the surfactant system, taking intraalveolar and intracellular surfactant together, is highly dynamic and in continuous flux. The intra-alveolar surfactant is usually turned over every 5–10 h (Wright and Dobbs 1991).

AE2 cells are critical in surfactant homeostasis and the regulation of the pool size and composition of the functional surfactant within the alveolar space. Hence, structural and functional alterations of AE2 cells and their LB are also linked with acute and chronic lung injury (Milos et al. 2017; Birkelbach et al. 2015; Haller et al. 2018). In the context of clinical lung transplantation, the size of LB in the donor lung correlated with post-operative desaturation and early complication rates. The larger the LB were, the higher was the risk for early complication suggesting that disturbances of intracellular surfactant pool are an indicator or even causal factor for pulmonary dysfunction in lungs at risk (Fehrenbach et al. 1998b).

Qualitative and quantitative abnormalities of the intra-alveolar surfactant are typical features of acute lung injury and result in severe alterations in alveolar micromechanics (Tabuchi et al. 2016; Schiller et al. 2001; Knudsen et al. 2018; Lutz et al. 2015; Schmidt et al. 2007). Mechanical ventilation of patients suffering from acute lung injury has been shown to aggravate lung injury, a condition termed ventilation-induced lung injury (VILI) (Slutsky and Ranieri 2013). One mechanism of VILI pathogenesis is based on heterogeneous ventilation and alveolar interdependence (Mead et al. 1970; Albert et al. 2019). Heterogeneous ventilation is induced by alveolar oedema due to vascular leak after injury of the blood–gas barrier but also results from surfactant dysfunction-mediated alveolar instability. Oedema-filled or collapsed alveoli exert tethering forces on adjacent, healthy alveoli and this causes local injurious overdistension (Albert et al. 2019; Perlman et al. 2011; Wu et al. 2014). Hence, pre-injured lungs with abnormalities of intra-alveolar surfactant are prone to VILI due to abnormal alveolar micromechanics including intratidal alveolar recruitment and derecruitment (= atelectrauma) as well as alveolar overdistension (= volutrauma). Both of these mechanisms trigger the progression of lung injury (Slutsky and Ranieri 2013), but can also propagate surfactant abnormalities (Albert 2012). In animal models of VILI using large tidal volumes during mechanical ventilation to simulate volutrauma, the conversion of active alveolar surfactant (so-called large aggregates) into inactive alveolar surfactant subtypes (so-called small aggregates) was increased and the composition of alveolar surfactant altered, e.g., increased fraction of cholesterol, so that the surfactant function was interrupted by mechanical ventilation (Veldhuizen et al. 1996; Veldhuizen et al. 2002; Vockeroth et al. 2010; Milos et al. 2017). These alterations of intra-alveolar surfactant predate the manifestation of VILI (Maruscak et al. 2008) that eventually leads to severe degradation of lung structure and function (Smith et al. 2020).

Within 1 day, intratracheal instillation of bleomycin leads to quantitative and qualitative surfactant abnormalities linked with microatelectases during mechanical ventilation with positive end-expiratory pressure (PEEP) below 5 cmH_2_O, so that these lungs can be considered as lungs at risk for propagation of surfactant dysfunction and injury progression during mechanical ventilation (Knudsen et al. 2018; Lutz et al. 2015). Hence, the present study investigates the effects of early bleomycin-induced lung injury in combination with mechanical ventilation with different PEEP levels on the ultrastructure of the blood–gas barrier and the AE2 cells including the intracellular surfactant pool. The PEEP level determines whether bleomycin-injured lungs exhibit microatelectases or not. Therefore, we opted to ventilate the lungs with PEEP = 1 cmH_2_O, where microatelectases are present, or at PEEP = 5 cmH_2_O, where microatelectases are nearly absent. Since mechanical ventilation of lungs suffering from microatelectases would induce alveolar overdistension due to alveolar interdependence (Albert et al. 2019, 2020; Knudsen et al. 2018), we hypothesize that PEEP = 1 cmH_2_O ventilation of bleomycin-injured lungs will exhibit abnormalities of intracellular surfactant pool and interstitial abnormalities that demarcate injury progression within alveolar septa.

## Materials and methods

### Subjects and study design

All animal experiments were approved by the “Niedersächsisches Landesamt für Verbraucherschutz und Lebensmittelsicherheit” (LAVES, Oldenburg, Lower Saxony, Germany, approval number 17/2068) which house the German equivalent of an institutional animal care and use committee, according to the European Animal Welfare Regulations. After randomization, male rats (Fisher 344/DuCrl, Charles River, Sulzfeld, Germany) were either treated intratracheally with Bleomycin (Group B; Dosage: 4.5 U/kg bodyweight, *n* = 20) or not (Group H, healthy, *n* = 17). This dosage of Bleomycin has been shown to result in subclinical lung injury at day 1, meaning that clinical presentation, oxygen saturation and tissue elastance measurements are not substantially different from healthy controls (Lutz et al. 2015; Knudsen et al. 2018; Albert et al. 2020). At day 1, animals were further randomized into three subgroups characterized by different mechanical ventilation protocols. The groups H/no-ventil (*n* = 6) and B/no-ventil (*n* = 6) were not ventilated and represented the baseline situation before entering mechanical ventilation. The other groups were invasively ventilated for 3 h with either PEEP = 1 cmH_2_O [groups H/PEEP1 (*n* = 5) and B/PEEP1 (*n* = 7)] or PEEP = 5 cmH_2_O [groups H/PEEP5 (*n* = 6) and B/PEEP5 (*n* = 7)]. The tidal volume was 10 ml/kg bodyweight room air, the respiratory rare 90/min and the inspiratory-to-expiratory ratio was 1:2. At the beginning of mechanical ventilation, and every 60 min during mechanical ventilation, recruitment maneuvers consisting of an increase of the airway opening pressure to 30 cmH_2_O were performed. After mechanical ventilation, two pressure-controlled pressure–volume (PV) loops were recorded to calculate the quasistatic compliance (Cst). For this purpose, the airway opening pressure was increased stepwise with a step size of 3.86 cmH_2_O from an onset airway opening pressure of 3–30 cmH_2_O. Afterwards, the pressure deceased with the same step size from 30 to 3 cmH_2_O. The displaced air volume was determined and using the Salazar–Knowles equation the slope of the PV loop at 5 cmH_2_O on expiration was computed to determine Cst (Salazar and Knowles 1964). The procedures regarding the mechanical ventilation were as follows: the rats were anesthetized with an intraperitoneal injection of Ketamine (80 mg/kg bodyweight) combined with Xylazine (5 mg/kg bodyweight). Afterwards, a tracheotomy was performed and the animals were connected to the newFlexiVent rodent ventilator (SCIREQ®, Montreal) and mechanically ventilated as described above. During mechanical ventilation narcosis was maintained by means of 1% isoflurane in room air combined with subcutaneous injection of 2 mg/kg bodyweight butorphanol (Torbugesic^®^).

### Tissue harvest and processing

After mechanical ventilation, rats were disconnected from the ventilator, the abdomen and chest wall were opened and the abdominal aorta incised. The pulmonary circulation was flushed with heparin (12,500 IU/L) in 0.9% sodium chloride solution via the right ventricle before the hilus of the left lung was clamped. The right lungs were lavaged with three portions of 4 ml 0.9% sodium chloride solution. Afterwards, the right hilus was ligated, the right lung removed and the tissue was cut in pieces and subjected to a smooth fractionator sampling to obtain several representative sets of tissue pieces (Gundersen 2002) for measurements of the expression of surfactant proteins B and C and Abca3 as detailed below. Immediately after the sampling process, the sampled tissue pieces were frozen in liquid nitrogen and stored in the − 80 °C freezer until further investigation. The left lungs were unclamped and fixed by airway instillation of 1.5% glutaraldehyde, 1.5% paraformaldehyde in 0.15 M HEPES buffer. During fixation a constant hydrostatic pressure of 25 cmH_2_O was used. After determination of the volume of the left lung by means of fluid displacement method (Scherle 1970), a systematic uniform random sampling was carried out based on established methods to obtain an unbiased and representative set of tissue for light and electron microscopic investigations of the left lung (Tschanz et al. 2014). The 3–5 tissue slices sampled for light microscopy were embedded in Technovit 8100 (Kulzer GmbH, Wehrheim, Germany), while samples for electron microscopy (at least 6 per lung) were embedded in epoxy resin (Epon^®^, Polyscience, Hirschberg an der Bergstr., Germany) based on established protocols (Mühlfeld et al. 2013). Technovit 8100-embedded tissue was sectioned into 1.5-µm-thick slices and stained with toluidine blue; while, epoxy resin-embedded material was cut into 80-nm-thick ultrathin sections. Two consecutive ultrathin sections were collected and placed on one grid to create a disector pair—the distance from the top of the first section to the top of the second section was 80 nm. This value was verified by measurements of folds in the ultrathin sections.

### Design-based stereological analyses

Quantitative morphology was performed according to the recommendations of the American Thoracic Society and European Respiratory Society (Hsia et al. 2010). At the light microscopic level, the newCAST software (Version 3.2, Visiopharm, Hoersholm, Denmark) was used. Light microscopy was used to determine the absolute volume of the interalveolar septa within the lung (*V*(sep,lung)) and the number-weighted mean volume of AE2 cells ($\nu_{N}$(AE2)). These two parameters represented the reference space for further electron microscopic studies. For this purpose, a systematic uniform random area sampling was applied to obtain a representative set of fields of view for detailed analyses distributed on all sampled tissue slices of an organ. For determination of *V*(sep,lung), test points were projected onto the sampled fields of view and test points hitting the interalveolar septa and the reference space were counted. The ratio of these two was the volume fraction of interalveolar septa within the reference space. Multiplication of the volume fraction and the lung volume (reference space) resulted in *V*(sep,lung). The number-weighted mean volume of AE2 cells was determined by means of the nucleator method where sampling of AE2 cells was based on the appearance of a nucleolus as a unique point within AE2 cells (Gundersen 1988; Knudsen et al. 2007). At the electron microscopic level the composition of the inter-alveolar septa, including the arithmetic mean thickness of the blood–gas barrier and the surface area of the alveolar epithelium and endothelium, was determined using single sections. An unbiased, representative set of images was sampled by means of systematic uniform random area sampling on 6 ultrathin sections per lung based on the *y*–*x* coordinates of the stage of the Morgagni transmission electron microscope (FEI, Eindhoven, The Netherlands) equipped with a Valeta digital camera (Olympus Soft Imaging Solutions, Münster, Germany). Using the Stepanizer online tool, a coherent test system of test points for point counting and intersection counting was superimposed on the sampled transmission electron microscopic images. Point counting was used to determine volume fractions of different structures within the interalveolar septa. Volume fractions were calculated as ratios of points hitting the structure of interest and the entirety of the reference space which was the volume of the interalveolar septa. The following structures were differentiated to calculate volume fractions within septal walls: alveolar epithelial cells (AE) including both the alveolar epithelial type 1 and AE2 cells (*V*_*V*_(AE/sep)), interstitial cells (*V*_*V*_(IC/sep)), extracellular matrix (*V*_*V*_(ECM/sep)), collagen fibrils (*V*_*V*_(col/sep)), extracellular matrix other than collagen fibrils (*V*_*V*_(resECM/sep)), endothelial cells (*V*_*V*_(endo/sep)) and capillary lumen (*V*_*V*_(caplumen/sep)). These volume fractions were consequently converted into absolute volumes within reference space to avoid the reference trap (Beike et al. 2019). In the results we will exclusively report the absolute volumes of the structures. In addition, test lines were used to quantify the air-covered surface area of AE1 cells (*S*(AE1_air,sep)) and AE2 cells (*S*(AE2_air,sep)) as well as the surface area of the endothelium (*S*(endo,sep)) on the blood side of the alveolocapillary barrier. The arithmetic mean thickness of the blood–gas barrier ($\tau$(bgb)) was calculated as a volume-to-surface ratio, including the volume of the blood–gas barrier and the surface area of this barrier on the air and blood side (Weibel and Knight 1964). As the composition of blood–gas barrier was known, the thickness of the different components, e.g., the alveolar epithelium ($\tau$(AE)), the interstitium including the interstitial cells as the extracellular matrix ($\tau$(inter)) and the endothelium ($\tau$(endo)) was calculated.

In a further step, the ultrastructure of the AE2 cells was investigated in more detail. This investigation was in part based on the physical disector (Sterio 1984) and for this purpose, two consecutive ultrathin sections of a thickness of 80 nm were mounted in parallel on one grid so that the distance from the top of the first to the top of the second section, the so-called disector height, was 80 nm (Beers et al. 2017). On both of these sections, all profiles of AE2 cells were completely imaged so that image pairs of the same AE2 cell with a distance of 80 nm from each other were obtained. On one of these sections, test points were projected and all test points hitting the AE2 cell were counted. Since each test point was associated with an area (area per point), the area of the profiles of AE2 cells could be calculated. The multiplication of this area with the disector height resulted in a test volume, the so-called disector volume, the pre-requisite for number estimations (Weibel et al. 2007). The image pairs showing profiles of identical AE2 cells were compared with each other. According to the physical disector counting principle, LBs present on one section but not on the other were counted. This number represented the number of newly emerged LB in the given volume so that the numerical density of LB per unit AE2 cell ($\nu_{N}$(AE2) = reference space) could be calculated (*N*_*V*_(LB/AE2). Since the reference volume, the number-weighted mean volume of AE2 cells was known, the numerical density could be converted into an absolute number of LB per AE2 cell (*N*(LB,AE2)) by multiplication with $\nu_{N}$(AE2). In addition, point counting was used to determine the volume fractions of those cellular organelles that are involved in surfactant homeostasis in AE2 cells. These included the LB, the electron-dense multivesicular bodies (d-mvb) and electron-lucent multivesicular bodies (l-mvb). At the end, the volumes of LB (V(LB,AE2), d-mvb (*V*(d-mvb,AE2) and l-mvb (*V*(l-mvb,AE2) per AE2 cell were determined. The number and volume per cell of LB were used to calculate the number-weighted mean volume of LB ($\nu_{N}$(LB)). The point sampling intercept method was applied to determine the volume-weighted mean volume of LB ($\nu_{v}$(LB)) (Gundersen and Jensen 1985). Due to the sampling procedure giving larger particles (in this case LB) a higher probability of being measured, the volume-weighted mean volume is an over representation of larger particles so that it is higher than the number-weighted mean volume. In mathematical terms the volume-weighted mean volume is dependent on the number-weighted mean volume and the coefficient of variation of particles sizes (Gundersen and Jensen 1985) so that we used in the present study $\nu_{N}$(LB) and $\nu_{v}$(LB) to calculate the coefficient of variation of LB sizes (CV($\nu_{x}$(LB)) for each study group.

Finally, the surface area of the apical (*S*(apical,AE2)) and the basolateral plasma membrane per AE2 cell was calculated. For this purpose, intersection counting of test lines with the apical and basolateral plasma membrane was performed at electron microscopic level. These two compartments could be separated from each other by the identification of the cell–cell contacts, e.g., the tight junctions, between alveolar epithelial cells. The reference space was the volume of the AE2 cells (Knudsen et al. 2011).

In general, 100–200 counting events per parameter were generated at electron microscopic level distributed on at least 100 fields of view from 6 samples per lung. This has been shown to result in a sufficient precision of stereological parameters (Gundersen and Jensen 1987; Mathieu et al. 1981).

### RT-PCR of surfactant proteins B and C and Abca3

Using ISOLATE II RNA Mini Kit (Bioline, Berlin, Germany), total cell RNA was isolated from the frozen tissue. The RNA concentration and purity was quantified in each sample using Nanodrop based on spectrometry. Afterwards, reverse transcription of 1 µg/µl RNA in cDNA was performed using iScript™ cDNA Synthesis Kit and a Thermocycler (C1000™ BioRad). The quantitative RT-PCR was based on the 2^△△CT^ – method (Livak and Schmittgen 2001). The primers are detailed in Table 1; the house-keeper gene was HPRT (Hypoxanthine Guanine Phosphoribosyltransferease). *N* = 4–5 independent measurements were performed per subgroup.

**Table 1:** Genes and primers

Gene	Proteine	Forward primer	Reverse primer	Referenzsequenz
Sfrpc	Surfactant protein C	AGATGGTCCTTGAGATGAGCA	ATACACAACGATGCCAGTGGA	NM_017342.2
Sfrpb	Surfactant protein B	ACACAGGACCTCTCTGAGCA	CCAGCACACCCTTGGGAATC	NM_138842.1
Abca3	ATP-binding cassette subfamily 1, member 3	GGCCTTCCTGCTGTGTTTTG	AGAAGTAAAGGAAGCCGCCC	XM_220219.9
Hprt1	Hypoxanthine phosphoribosyltransferase 1	ACAGGCCAGACTTTGTTGGA	TCCACTTTCGCTGATGACACA	NM_012583.2

### Expression of surfactant proteins B and C in lung homogenate at protein level

Since we were interested in the intracellular surfactant pool, we used tissue homogenate after broncho-alveolar lavage to identify the precursor proteins of SP-B (proSP-B) and SP-C (proSP-C) which are usually found in AE2 cells but not within the alveolar space. The Western blot measurements of proSP-B and proSP-C were performed according to the published methods (Lopez-Rodriguez et al. 2016; Korfei et al. 2008). In brief, frozen tissue pieces from the smooth fractionator sampling process were homogenized and centrifuged (12,000 rpm, 20 min, 4 °C), and total protein content in the supernatant was determined using BCA protein assay (Thermo Scientific, Waltham, Ma, USA). 10 µg of proteins was transferred into pockets of 12% polyacrylamide gels. Afterwards, an electric field of 180 V was created for 2 h to separate proteins according to molecular weight and charge by gel electrophoresis. For visualization, proteins were transferred to polyvinylidene fluoride (PVDF) membrane, blocked with bovine serum albumin (BSA) and incubated at 4 °C overnight with the primary antibody for pro SP-B at a dilution of 1:2000 (polyclonal rabbit anti-proSP-B, Merck, AB3430) and for proSP-C at a dilution of 1:1000 (polyclonal rabbit anti-proSP-C, Merck AB3786). After washing with TBS-T (TBS-0.1% Tween 20), the secondary antibody (HRP-conjugated polyclonal swine anti rabbit, DAKO, Glostrup, Denmark) was added for 1 h at room temperature using a dilution of 1:2000 for proSP-C and 1:5000 for proSP-B. β-actin was used as housekeeper protein and the primary antibody was also incubated over night at 4 °C at a dilution of 1:2000. Each gel could be run with 12 lanes and blots were repeated 2 times for proSP-B and 3 times for proSP-C. The ImageLab software was used to quantify the intensity of the bands. The intensity measured in proSP-B and proSP-C bands was related to the intensity of the housekeeper, the β-actin. For proSP-C, we quantified two peptides at 21 kD and 16 kDa (Vorbroker et al. 1995). These ratios were normalized to the measurement in not ventilated healthy controls (group H/no_ventil) in which the expression was accordingly 1.

### Statistics

*GraphPad Prism Version 7* (GraphPad Software, San Diego, California, USA) was used for statistical assessment. Since the factors “pretreatment” (with bleomycin) and the mechanical ventilation (vent: no nentilation vs. PEEP 1 vs. PEEP 5) were considered to have an effect on the composition of the blood–gas barrier and the ultrastructure of AE2 cells, a two-way ANOVA was used to take these influencing factors into account. In case of significant differences based on the two-way ANOVA the Tukey post hoc test for multiple comparisons and adjustment of the *p* level was added. Pearson correlation coefficient was determined to investigate the link between Cst, thickness of the blood–gas barrier as marker of lung injury and abnormalities of AE2 cells. In general, p values below 0.05 were considered as statistically significant.

## Results

### Mechanical ventilation and the blood–gas barrier

**Fig. 1:**
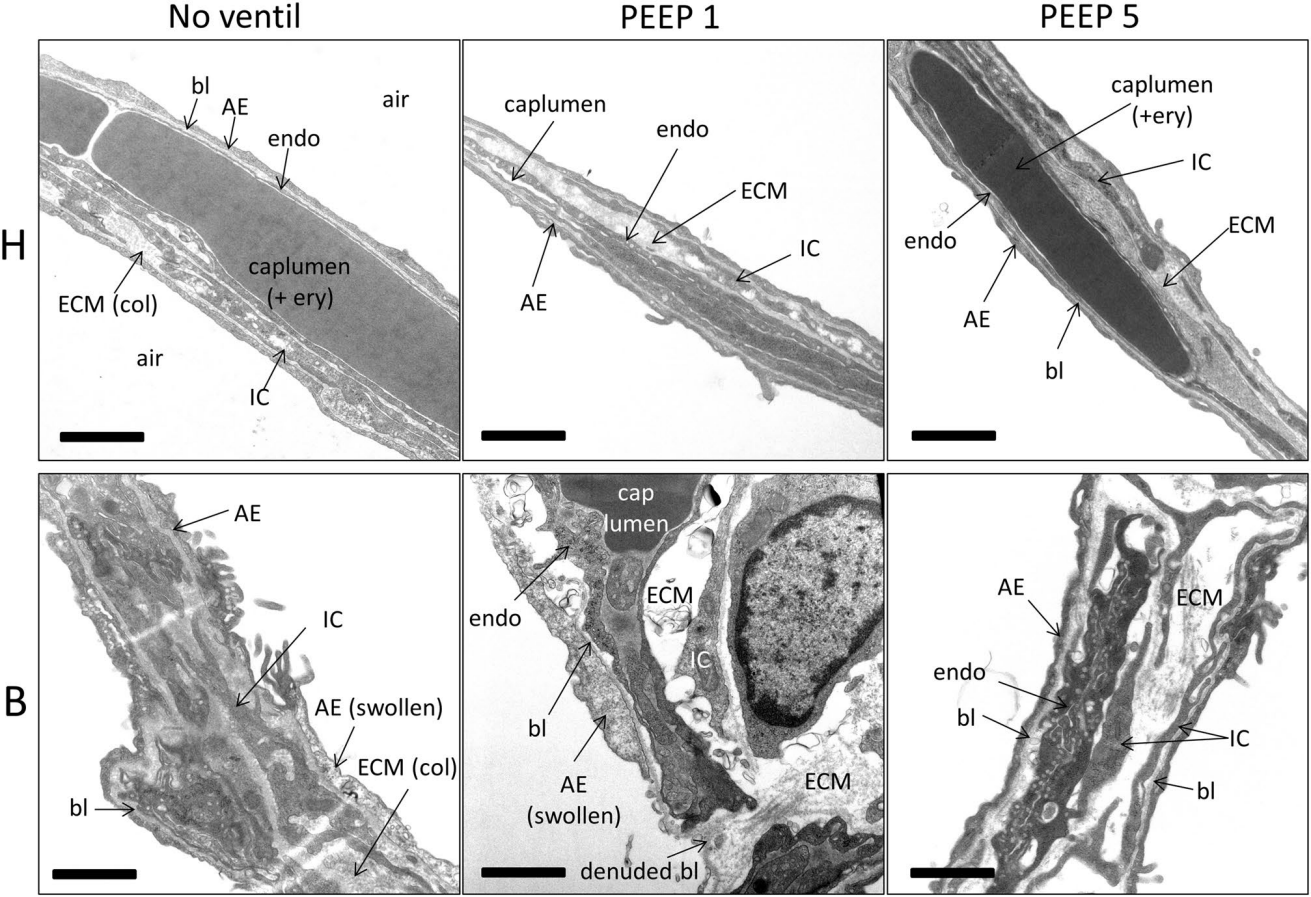
Ultrastructure of the blood–gas barrier. In healthy controls (H) the ultrastructural presentation of the blood–gas barrier consisting of the alveolar epithelium (AE), the interstitium and the endothelium (endo) was unaffected by ventilation. After bleomycin challenge (B) however, signs of injury of the alveolar epithelial (AE) cells could be found. AE cells were either swollen (characterized by cytoplasmic clearance) or completely missing so that the basal lamina (bl) was denuded. In healthy controls, the interstitium, consisting of extracellular matrix (ECM) and interstitial cells (IC) is thin and compact; while after bleomycin challenge, there is widening in some areas. Further abbreviations: *col* collagen fibrils, *air* alveolar airspace, *caplumen* capillary lumen, *ery* erythrocyte. Scale bar: 1 µm

Qualitative investigations of the interalveolar septa demonstrated thin blood–gas barriers throughout the healthy (H) study groups, independent of mechanical ventilation or not (Fig. 1). The alveolar epithelial cells were slim at the thin side of the blood–gas barrier with hardly any signs of injury such as clearance of the cytoplasmic ground substance, blebbing or fragmentation. The same was the case regarding the endothelial cells. On the thick side of the blood–gas barrier, which usually contributes 50% of the blood–gas barrier and where the epithelial and endothelial basal laminae are separated by interstitial tissue, the layers of the blood–gas barrier were also compact without any signs of an interstitial oedema. In contrast, epithelial injury could occasionally be observed in all 3 bleomycin groups (B). In B/no_ventil, swollen alveolar epithelial cells were seen on both the thin and the thick side of the blood–gas barrier. The interstitium between endothelial cells and epithelial cells on the thick side did not substantially differ from the healthy lungs and could be described as being relatively compact. The endothelial cells did not show any ultrastructural signs of injury. After mechanical ventilation with PEEP = 1 cmH_2_O in the bleomycin-injured lungs, denudations of the epithelial basal lamina were the most severe ultrastructural feature of injury. Also, the interstitial tissue between epithelial and endothelial basal laminae was widened and the collagen fibrils appeared to be torn apart and therefore less compact. Also in B/PEEP5, widening of the interstitial space within interalveolar septa could be observed. However, these abnormalities were by far not that severe as in B/PEEP1 and denudations of the epithelial basal lamina were virtually absent.

**Fig. 2:**
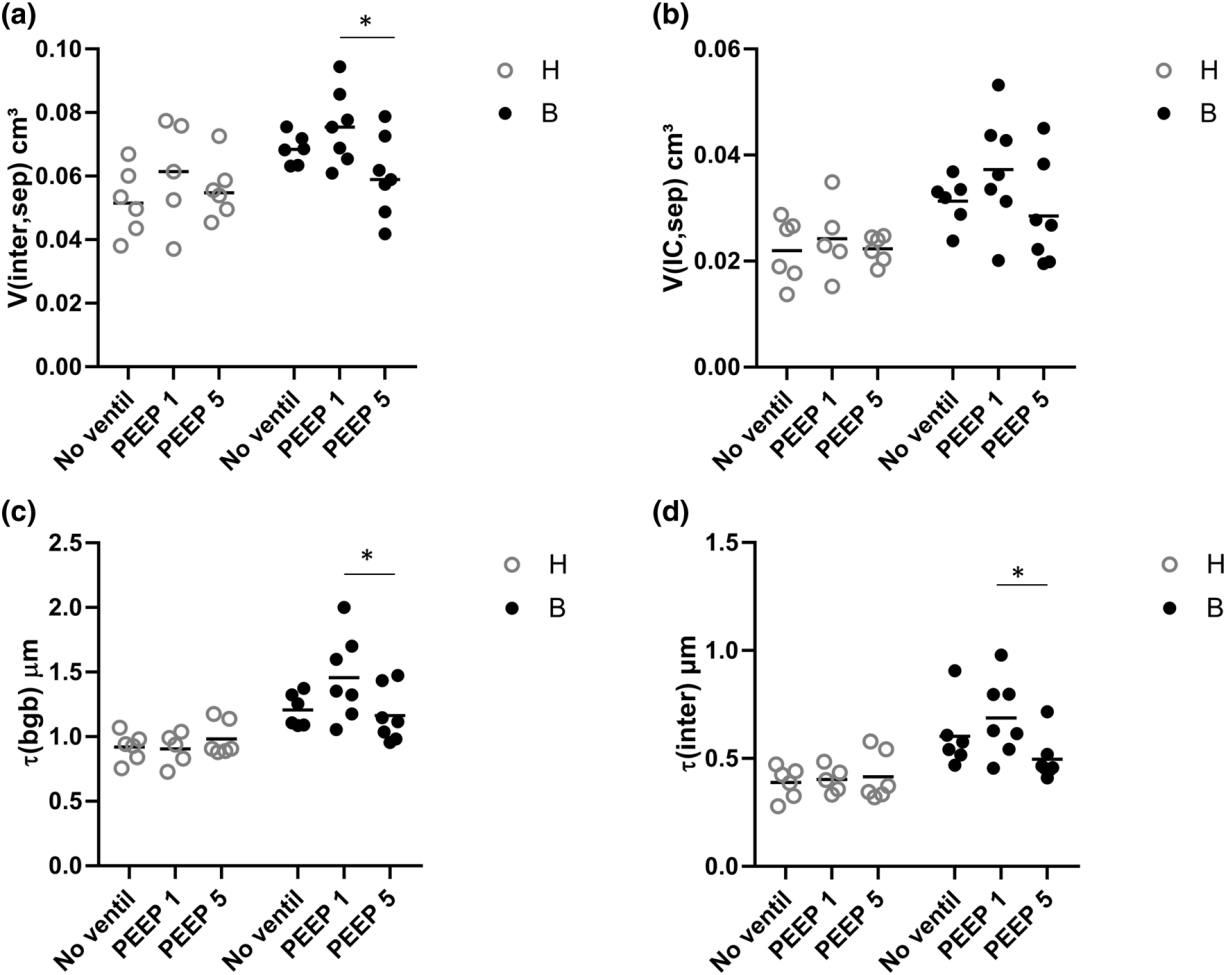
Stereological data characterizing abnormalities of the blood–gas barrier. Bleomycin challenge increases the absolute volume of the interstitium within the interalveolar septa (**a**), in particular after mechanical ventilation with PEEP = 1 cmH_2_O. The increase in V(inter,sep) is predominantly attributable to an increase in the volume of interstitial cells (**b**) and results in an increase in the total arithmetic mean thickness of blood–gas barrier (**c**). In (**d**) the arithmetic mean thickness of the interstitium is shown. Statistical analyses are based on a two-way ANOVA taking the factors “bleomycin pre-treatment” and “mechanical ventilation” into account. Statistically significant differences after adjustment of the *p* level for multiple testing using Tukey correction is indicated as follows:*p < 0.05

Based on these qualitative observations, we quantified the composition of the interalveolar septa and the thickness of the blood–gas barrier. The data are summarized in Table 2 and visualized in part in Fig. 2. The statistical analyses took the factors bleomycin pre-treatment and the mechanical ventilation into consideration. Neither bleomycin pre-treatment nor mechanical ventilation had a significant effect on the volume of AE cells within interalveolar septa, *V*(AE,sep). The surface area of air-covered AE1 cells, *S*(AE1,sep), was reduced by bleomycin pre-treatment; while ventilation did not show an effect. The surface area of AE2 cells covered by air *S*(AE2,sep) was unaffected by bleomycin pre-treatment and mechanical ventilation. The absolute volume of the interstitium within interalveolar septa was significantly increased due to bleomycin pre-treatment (Table 2). The adjustment of the *p* value by means of Tukey post hoc test illustrated a significantly higher V(inter,sep) in B/PEEP1 compared to B/PEEP5; while, there were no differences between B/no-ventil and B/PEEP5 (Fig. 2a). Since the interstitium consists of interstitial cells (IC) and extracellular matrix (ECM) which can further be distinguished in collagen fibrils (col) and ECM other than collagen fibrils (restECM), the volumes of these components were analyzed further. Neither bleomycin pre-treatment nor mechanical ventilation had an effect on *V*(ECM,sep) (Table 2). On the other hand, bleomycin-induced lung injury resulted on day 1 after instillation already in an increase in the volume of interstitial cells (Fig. 2b; *V*(IC,sep)) and this increase was most pronounced in B/PEEP1 so that the increase in *V*(inter,sep) at the organ scale was primarily a consequence of an increase in the volume of interstitial cells within septa rather than an increase in the volume of extra cellular matrix. Finally, the capillary network within the interalveolar septa was analyzed. This included the quantification of the volumes of endothelial cells (*V*(endo,sep)) and capillary lumen (*V*(caplumen,sep)) as well as the surface area of the endothelial cells (*S*(endo,sep)). None of these parameters was affected by bleomycin pretreatment or mechanical ventilation. The volume of the blood–gas barrier and its surface areas at the air and blood side were used to determine the arithmetic mean thickness of the blood–gas barrier $\tau$(bgb). $\tau$(bgb) was increased as a result of the bleomycin pre-treatment and this increase was most pronounced in B/PEEP1 so that the Tukey post hoc test for adjustment of the *p* level revealed that, within the B group, $\tau$(bgb) was significantly higher in B/PEEP1 compared to B/PEEP5 (Fig. 2c). Dividing the blood–gas barrier into its components, alveolar epithelium, interstitium and endothelium, reveals that the increase in barrier thickness was mainly a result of the increase in the thickness of the interstitial layer $\tau$(inter). This alteration was most pronounced in the bleomycin groups where $\tau$(inter) was elevated in B/PEEP1 compared to B/PEEP5 (Fig. 2d). With regard to the thickness of the endothelial layer $\tau$(endo), there was a significant effect assigned to the bleomycin pretreatment but the Tukey post hoc test did not reveal differences between the subgroups.

**Table 2:** Composition of the interalveolar septa

Parameter	H			B			2-way ANOVA		
	No ventil	PEEP1	PEEP5	No ventil	PEEP1	PEEP5	Bleo	Vent	inter
*V*(AE,sep) mm^3^	22.1 (6.7)	24.3 (8.1)	26.5 (6.1)	18.4 (5.5)	23.7 (8.6)	27.1 (14.4)	0.690	0.208	0.840
*S*(AE1_air, sep) cm^2^	976 (250)	935 (461)	1041 (276)	752 (97)	777 (238)	814 (193)	**0.02**	0.696	0.984
*S*(AE2_air,sep) cm^2^	39.2 (12.9)	39.1 (14.3)	55.5 (49.7)	22.2 (15.4)	55.6 (32.1)	76.8 (55.1)	0.679	0.058	0.402
*V*(inter,sep) mm^3^	52.0 (10.6)	60.9 (16.8)	56.0 (9.4)	68.5 (4.8)	75.5 (12.8)	60.1 (12.8)^#^	**0.004**	0.087	0.35
*V*(IC,sep) mm^3^	22.0 (6.0)	24.2 (7.2)	22.3 (2.6)	31.3 (4.5)	37.3 (10.6)	28.5 (9.7)	** < 0.001**	0.207	0.536
*V*(ECM,sep) mm^3^	30.0 (6.0)	36.7 (11.0)	33.7 (7.9)	37.2 (5.1)	38.2 (1.0)	31.5 (5.4)	0.337	0.284	0.337
*V*(col,sep) mm^3^	11.2 (2.5)	11.3 (4.7)	10.7 (2.5)	13.2 (5.3)	12.2 (3.1)	10.4 (3.6)	0.772	0.522	0.772
*V*(resECM,sep) mm^3^	18.8 (4.0)	25.3 (6.0)	23.0 (6.0)	24.0 (2.7)	26.0 (7.3)	21.1 (4.0)	0.466	0.133	0.266
*V*(endo,sep) mm^3^	24.1 (6.3)	23.8 (4.0)	25.1 (5.5)	25.9 (5.8)	23.8 (8.8)	20.7 (5.6)	0.537	0.666	0.491
S(endo,sep) cm^2^	1147 (289)	1383 (517)	1132 (159)	1110 (197)	1013 (355)	979 (264)	0.057	0.634	0.321
*V*(caplumen,sep) cm^3^	19.2 (7.4)	21.1 (7.4)	16.4 (3.4)	18.1 (1.7)	16.0 (4.9)	14.8 (7.2)	0.289	0.227	0.875
$\tau$(bgb) µm	0.91 (0.12)	0.91 (0.13)	1.21 (0.33)	1.21 (0.13)	1.46 (0.33)	1.16 (0.21)^#^	** < 0.001**	0.281	0.074
$\tau$(AE) µm	0.20 (0.03)	0.20 (0.02)	0.24 (0.03)	0.20 (0.12)	0.28 (0.11)	0.29 (0.14)	0.112	0.167	0.388
$\tau$(inter) µm	0.39 (0.07)	0.40 (0.06)	0.42 (0.11)	0.60 (0.16)	0.69 (0.18)	0.50 (0.10)^#^	** < 0.001**	0.224	0.134
$\tau$(endo) µm	0.22 (0.03)	0.20 (0.04)	0.23 (0.03)	0.27 (0.04)	0.25 (0.03)	0.22 (0.05)	**0.022**	0.29	0.172

Bold values highlight statistically significant effects of the factors (*p* < 0.05)

Summarized stereological data are given as mean (standard deviation). A two-way ANOVA on ranks was performed with the factors of treatment (bleomycin effects, B vs. H) and ventilation (no_ventil vs. PEEP1 vs. PEEP5). An adjustment of the *p* levels for multiple testing was applied using the Tukey post hoc test

*V* volume, *S* surface area, $\tau$ arithmetic mean thickness, *AE1* alveolar epithelial type I cell, *sep* interalveolar septum, *AE1_air* alveolar epithelial type I cell covered by air, *AE2* alveolar epithelial type 2 cell, *AE2_air* alveolar epithelial type II cell covered by air, *inter* interstitium, *IC* interstitial cell, *ECM* extracellular matrix; col: collagen fibrils, *resECM* ECM other than collagen fibrils, *endo* endothelial cell, *caplumen* lumen of capillary network, *bgb* blood–gas barrier, *AE* alveolar epithelium taking type I and type II cells together

Within each group (B and H) statistical significant differences of ventilation effects are indicated as follows: *p < 0.05 vs. no ventil; ^#^*p* < 0.05 vs. PEEP1

### Alveolar epithelial type II cells and the intracellular surfactant pool

**Fig. 3:**
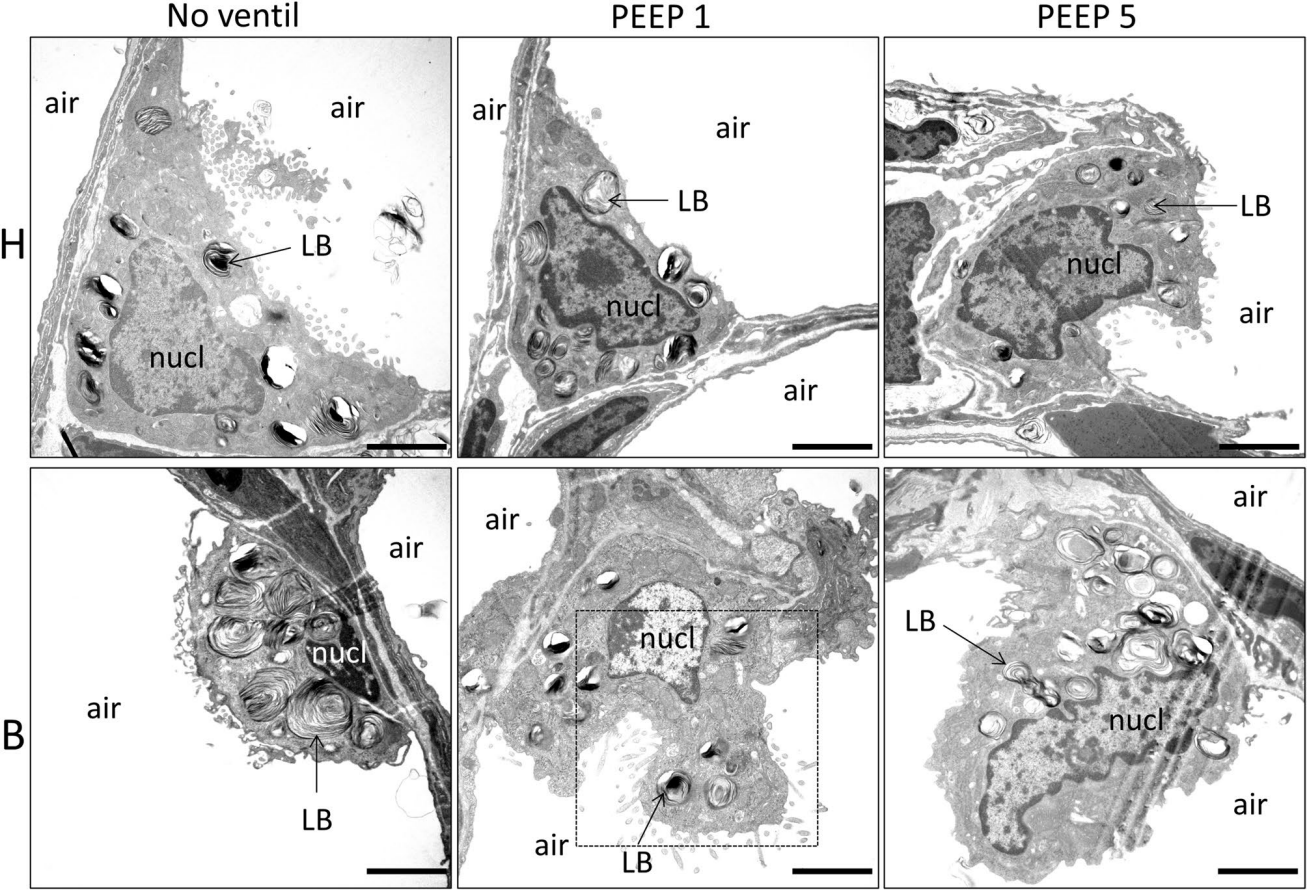
Representative ultrastructure of AE2 cells in healthy (H) and bleomycin (B)-pre-injured lungs subjected to different ventilation. In B/no_ventil, some AE2 cells were characterized by very large LB compared to H/no_ventil. The dashed line illustrates the region of the AE2 cell which is enlarged in Fig. 4. *LB* lamellar body, *air* alveolar airspace, *nucl* nucleus. Scale bar: 2 µm

**Fig. 4:**
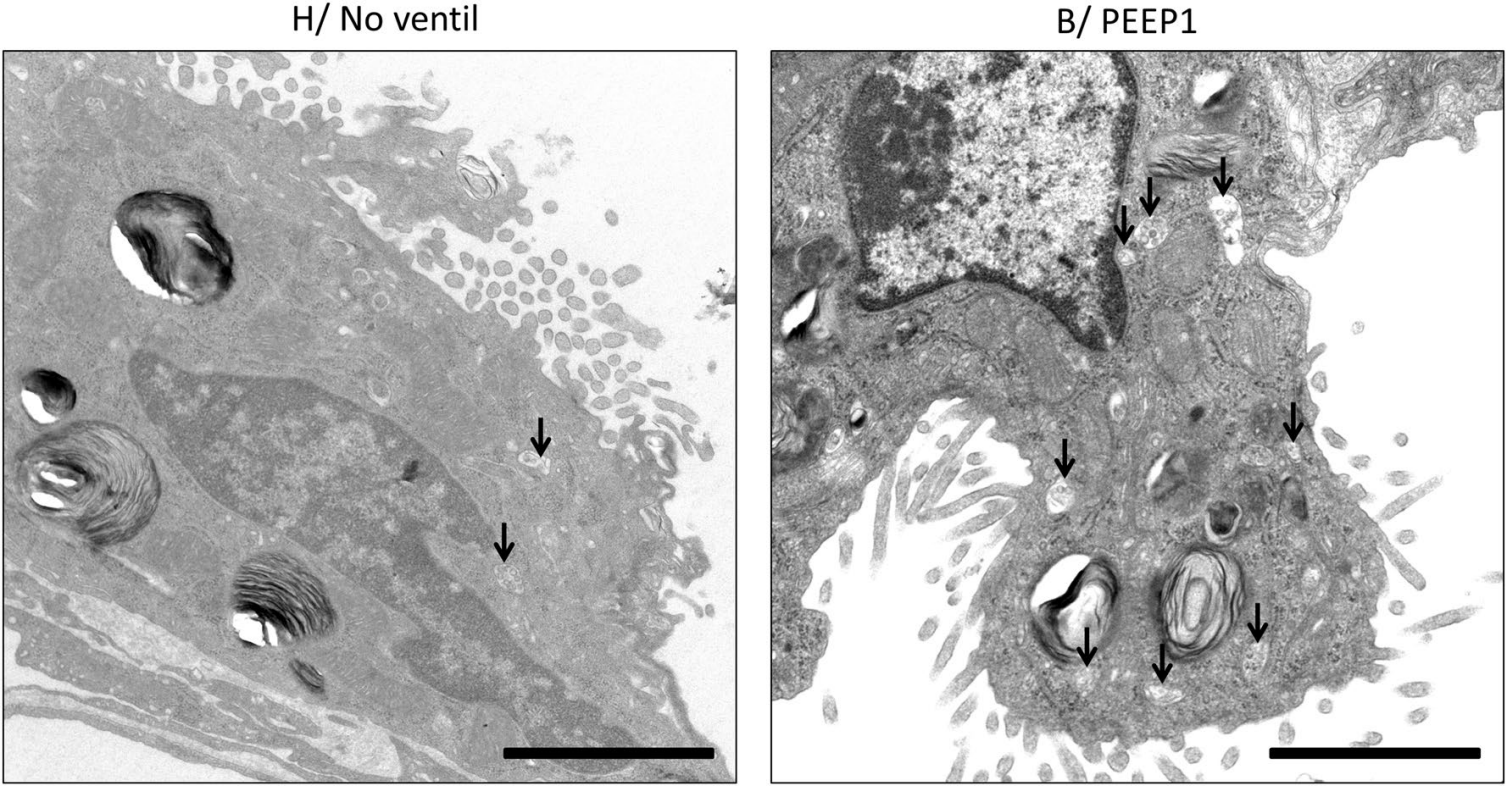
Electron-lucent multivesicular bodies, l-mvb, (arrows) were observed close to the apical plasma membrane. In B/PEEP1 some AE2 cells had a considerably high number of l-mvb compared to the other groups, e.g., H/no_ventil. B/ PEEP1 is a higher magnification from the AE2 cell illustrated in Fig. 3. Scale bar: 2 µm

Figures 3 and 4 demonstrate qualitative findings of AE2 cells from the different study groups. In general, after bleomycin challenge, there were some AE2 cells in which LB appeared to be abnormally enlarged (Fig. 3). A clear ventilation effect on the descriptive ultrastructure of the LB was not visible. Regarding the multivesicular bodies (mvb), the typical distribution of dense (d-) mvb and electron lucent (l-) mvb could be observed independent of pre-treatment as well as mechanical ventilation. The l-mvb were closer to the apical membrane, while the d-mvb appeared to be rather in the basal half of the cell. Multivesivular bodies were in all the study groups quite rare and small organelles. Nevertheless, some AE2 cells could be identified in B/PEEP1 in which profiles of l-mvb were quite frequently seen (Fig. 4, arrows). However, in general, no clear differences in the mvb compartment could be identified between the study groups. Hence, a quantification of the intracellular surfactant pool was performed using design-based stereological methods. This quantification also included the mvb which are involved in surfactant biosynthesis, recycling and degradation pathways.

**Fig. 5:**
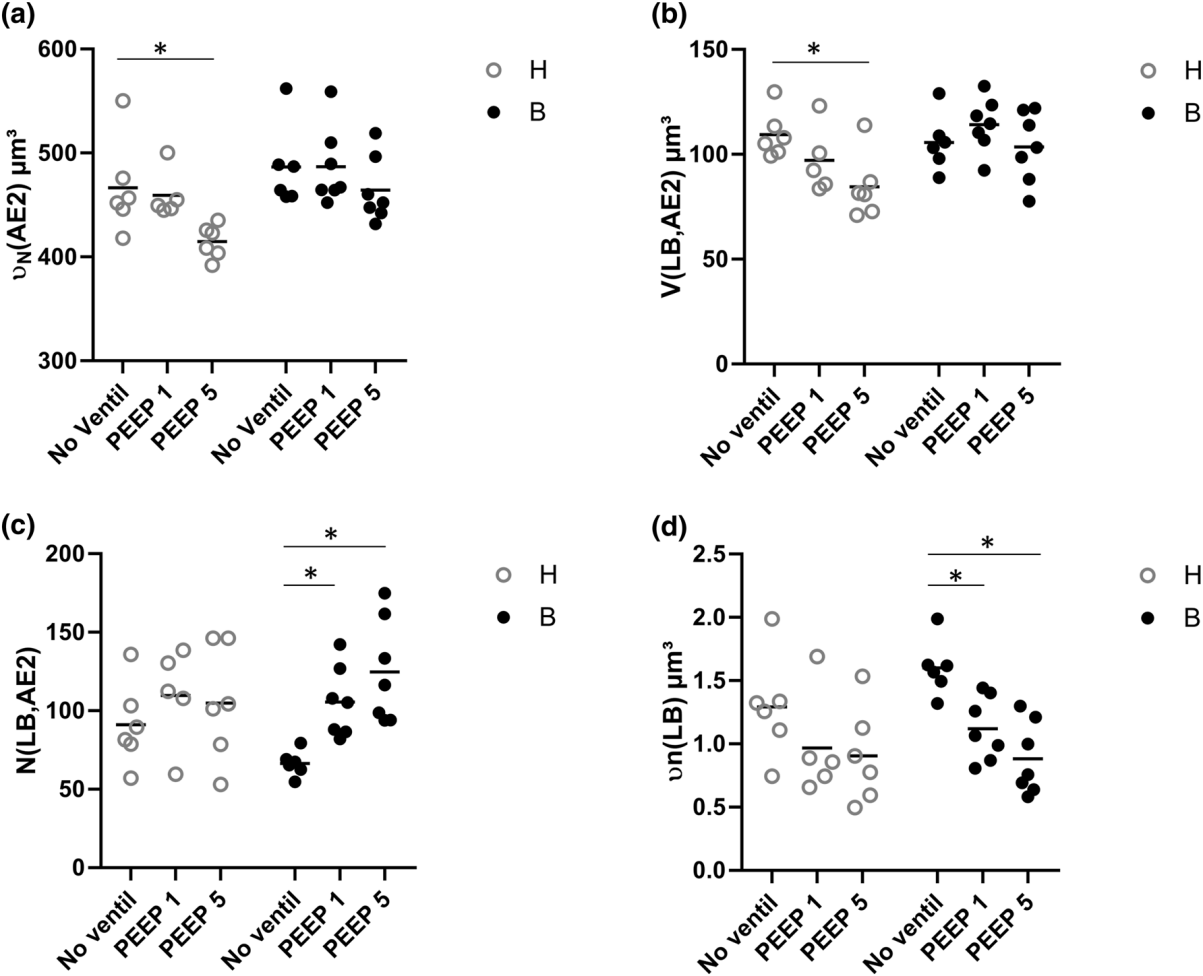
Stereological data characterizing AE2 cells and the intracellular surfactant pool. The number-weighted mean volume of the AE2 cells decreased with increasing PEEP during mechanical ventilation in healthy lungs but not in bleomycin-pre-injured lungs (**a**). In healthy lungs, mechanical ventilation was linked with a decrease in the volume of LB per AE2 cell a behavior which was not observed after bleomycin challenge (**b**). The number of LB per AE2 cell remained stable in healthy lungs but increased in bleomycin-pre-injured lungs after mechanical ventilation (**c**). In general, mechanical ventilation with increasing PEEP was associated with a decrease in the number-weighted mean volume of LB, a behavior which was much more pronounced in bleomycin-pre-injured lungs (**d**). Statistical analyses are based on a two-way ANOVA taking the factors “bleomycin pre-treatment” and “mechanical ventilation” into account. Statistically significant differences after adjustment of the *p* level for multiple testing using Tukey correction is indicated as follows:*p < 0.05

**Fig. 6:**
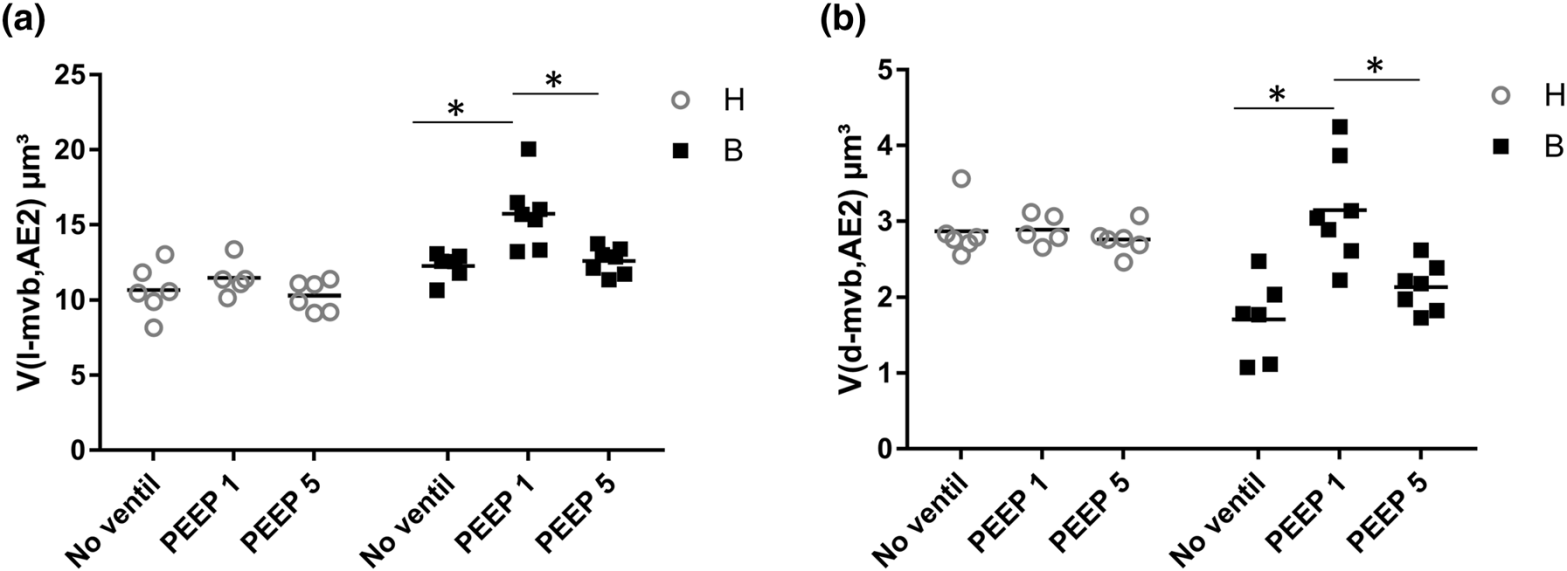
Stereological data related to multivesicular bodies (mvb). **a** The volumes of electron-lucent multivesicular bodies (l-mvb) per AE2 cell are largest after bleomycin challenge and mechanical ventilation with PEEP = 1 cmH_2_O. In (**b**) the volumes of electron-dense multivesicular bodies (d-mvb) are illustrated. Statistical analyses are based on a two-way ANOVA with the factors “bleomycin pre-treatment” and “mechanical ventilation”. Statistical significant differences after adjustment of the *p* level for multiple testing using Tukey correction is indicated as follows:*p < 0.05

The stereological data regarding AE2 cells and the intracellular surfactant pool are summarized in Table 3 and visualized in part in Figs. 5 and 6. The nucleator method was used to determine the number-weighted mean volume of AE2 cells ($\nu_{N}$(AE2)). $\nu_{N}$(AE2) was influenced by both bleomycin pre-treatment and mechanical ventilation (Table 3). Significantly larger mean sizes of AE2 cells were found after bleomycin challenge compared to healthy control. Moreover, Fig. 5a illustrates that in healthy lungs after mechanical ventilation with PEEP = 5 cmH_2_O, the size of AE2 cells was significantly smaller compared to H/no-ventil so that in healthy lungs, there was a clear effect on AE2 cell size assigned to mechanical ventilation. In bleomycin-pre-injured lungs, however, mechanical ventilation showed hardly any effects on $\nu_{N}$(AE2) (Fig. 5a). Hence, with increasing PEEP level from 1 to 5 cmH_2_O, the difference in the AE2 cell sizes became more pronounced between healthy lungs (H/PEEP5) and pre-injured lungs (B/PEEP5) (Fig. 5a). In a next step of the cascade sampling design (Ochs 2006) the AE2 cells were the reference space of the stereological investigations. The morphometric characteristics of the LB were investigated in terms of their absolute volume (*V*(LB,AE2)) and number (*N*(LB,AE2)) per AE2 cell as well as number-weighted mean size $\nu_{N}$(LB) and size variability. In healthy lungs, *V*(LB,AE2) decreased significantly with increasing PEEP during mechanical ventilation; while such a decrease could not be observed in bleomycin-pre-treated lungs (Fig. 5b). As a result *V*(LB,AE2) was significantly larger in the B groups compared to the H groups and within the H groups *V*(LB,AE2) was significantly smaller in H/PEEP5 compared to H/no_ventil. From these observations, the question arises whether the decrease in *V*(LB,AE2) with increasing PEEP level during mechanical ventilation was a consequence of a decrease in the number of LB per AE2 cell or a decrease in the mean size or both. The physical disector was used to determine the number of LB per AE2 cell *N*(LB,AE2). In healthy lungs, *N*(LB,AE2) remained roughly stable during mechanical ventilation with the investigated PEEP levels (Fig. 5c); while, there was a trend for a reduction in the mean size of LB ($\nu_{N}$(LB)) (Fig. 5d). In bleomycin-pre-treated lungs, however, N(LB,AE2) was smaller in B/no_ventil compared to the other groups including H/no_ventil. With mechanical ventilation of bleomycin-pre-treated lungs, however, N(LB,AE2) significantly increased and this increase was most pronounced with PEEP = 5 cmH_2_O ventilation. In general, mechanical ventilation had an effect on $\nu_{N}$(LB) and there was a trend for smaller LB in mechanically ventilated compared to non-ventilated healthy lungs (Fig. 5d). Such a ventilation-induced reduction in $\nu_{N}$(LB) was much more visible in bleomycin-pre-treated lungs where significantly reduced sizes were observed in B/PEEP5 and B/PEEP1 compared to B/no_ventil. The volume-weighted mean volume of lamellar bodies ($\nu_{v}$(LB)) was affected by the pre-treatment and demonstrated in general significantly larger values in the bleomycin-pre-treated lungs (Table 3). However, mechanical ventilation did not have an effect on $\nu_{v}$(LB). $\nu_{v}$(LB) was determined by the point sampled intercept method which means that larger LB have a higher probability of being analyzed than smaller ones so that in $\nu_{v}$(LB) larger LB are overrepresented. As such, $\nu_{v}$(LB) is directly dependent on $\nu_{N}$(LB) and the coefficient of variation of the LB volumes (CV($\nu_{X}$(LB)) (Gundersen et al. 1988). The fact that during mechanical ventilation with increasing PEEP, $\nu_{N}$(LB) decreased while $\nu_{v}$(LB) remained roughly stable in particular in the bleomycin groups can be explained by an increase in CV($\nu_{X}$(LB)) and, therefore, a higher variability on the sizes of LB. With this regard, in the bleomycin group CV($\nu_{X}$(LB) increased from 0.27 in B/no-ventil to 0.89 in B/PEEP5; while in the healthy group this increase was less pronounced from 0.29 in H/no-ventil to 0.70 in H/PEEP5.

**Table 3:** Intracellular surfactant

Parameter	H			B			2-way ANOVA		
	No ventil	PEEP1	PEEP5	No ventil	PEEP1	PEEP5	Bleo	Vent	inter
$\nu_{N}$(AE2) µm^3^	467 (45)	459 (23)	415 (16)*	486 (39)	487 (37)	464 (32)	**0.007**	**0.019**	0.533
*V*_V_(LB/AE2) [%]	23.5 (1.5)	21.1 (2.6)	20.4 (3.5)	21.7 (1.9)	23.6 (3.5)	22.3 (3.2)	0.148	0.499	0.354
*V*(LB,AE2) µm^3^	109 (11)	97 (16)	84 (16) *	106 (13)	114 (14)	104 (17)	**0.032**	0.053	0.113
*N*(LB,AE2)	91 (27)	110 (31)	105 (37)	66 (8)	106 (17)*	125 (33)*	0.748	**0.008**	1.155
$\nu_{N}$(LB) µm^3^	1.29 (0.41)	0.97 (0.41)	0.91 (0.38)	1.60 (0.22)	1.12 (0.23)*	0.88 (0.29)*	0.189	** < 0.001**	0.462
$\nu_{v}$(LB) µm^3^	1.40 (0.77)	1.26 (0.55)	1.35 (0.48)	1.72 (0.58)	1.91 (0.57)	1.59 (0.50)	**0.041**	0.868	0.645
*V*(mvb,AE2) µm^3^	13.5 (1.9)	14.3 (1.3)	12.8 (1.0)	14.0 (1.0)	18.9 (2.7)*	14.7 (0.9)^#^	** < 0.001**	** < 0.001**	**0.015**
*V*(l-mvb,AE2) µm^3^	10.7 (1.7)	11.4 (1.2)	10.1 (0.9)	12.3 (0.9)	15.7 (2.3)*	12.6 (0.9)^#^	** < 0.001**	** < 0.001**	0.081
*V*(d-mvb,AE2) µm^3^	2.9 (0.4)	2.9 (0.2)	2.8 (0.2)	1.7 (0.5)	3.1 (0.7)*	2.1 (0.3)^#^	**0.01**	** < 0.001**	**0.002**
*S*(PM,AE2) µm^2^	409 (76)	405 (34)	336 (32)	302 (48)	363 (107)	340 (96)	0.058	0.335	0.194
*S*(apical,AE2) µm^2^	150 (26)	159 (8)	131 (15)	142 (22)	163 (53)	161 (48)	0.451	0.487	0.379
*S*(bl,AE2) µm^2^	259 (55)	246 (36)	205 (18)	159 (28)	199 (55)	179 (48)	** < 0.001**	0.24	0.114

Bold values highlight statistically significant effects of the factors (*p* < 0.05)

Summarized stereological data are given as mean (standard deviation). A two-way ANOVA on ranks was performed with factors for treatment (bleomycin effects, B vs. H) and ventilation (no ventil vs. PEEP1 vs. PEEP5). An adjustment of the *p* level for multiple testing using the Tukey post hoc test was applied

$\nu_{N}$ number-weighted mean volume, *AE2* alveolar epithelial type II cell, *N* number, *V*_*V*_ volume fraction, *LB* lamellar body, $\nu_{v}$ volume-weighted mean volume, *l-mvb* electron-lucent multivesicular bodies, *d-mvb* dense multivesicular bodies, *S* surface area, *PM* cellular plasma membrane, *apical* apical plasma membrane, *bl* basolateral plasma membrane

Within each group (B and H) statistical significant differences of ventilation effects are indicated as follows: *p < 0.05 vs. no ventil; ^#^*p* < 0.05 vs. PEEP1

Based on the ultrastructural criteria, multivesicular bodies (mvb) can be divided into two groups, the electron lucent mvb (l-mvb) and the dense mvb (d-mvb). Since it has been suggested that these two cellular compartment fulfill important functions in surfactant homeostasis (Rooney et al. 1994; Williams 1984), the absolute volumes of l-mvb (*V*(l-mvb,AE2)) and d-mvb (*V*-(d-mvb,AE2) per AE2 cell were determined. In general, both bleomycin challenge and mechanical ventilation had an effect on the volumes of mvb and their subtypes (Table 3). Mechanical ventilation with PEEP = 1 cmH_2_O after bleomycin-induced lung injury was linked with a significant increase in V(l-mvb,AE2) compared to B/no_ventil, a behavior which could not be demonstrated with PEEP = 5 cmH_2_O ventilation (Fig. 6a). A similar behavior was observed for the volumes of d-mvb per AE2 cell (Fig. 6b). In healthy lungs, mechanical ventilation had a negligible effect on the volumes of l-mvb or d-mvb per AE2 cell.

The AE2 cells have a typical apical differentiation and it has previously been shown that an increased exocytosis activity of LB results in an increase in the surface area of the apical membrane (Knudsen et al. 2011). Therefore, we determined the apical as well as basolateral surface area per AE2 cell in our experimental groups. While no significant effects of bleomycin pretreatment or mechanical ventilation could be detected on the apical surface area *S*(apical,AE2), the surface area of the basolateral membrane *S*(bl,AE2) was significantly larger in H compared to B.

**Fig. 7:**
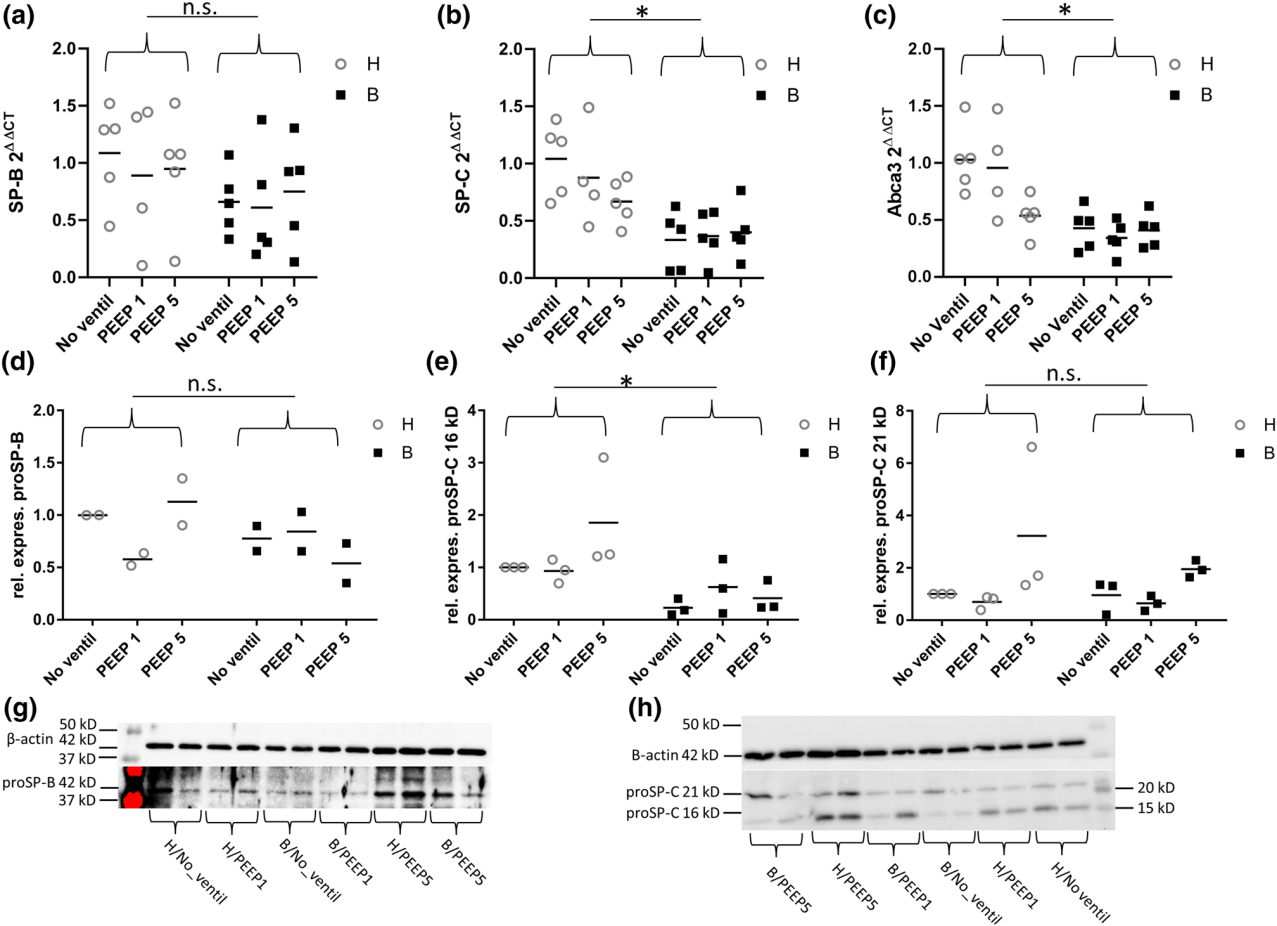
Expression of hydrophobic surfactant proteins B and C as well as lipid transporter Abca3. While bleomycin had no effect on SP-B RNA expression (**a**), both SP-C (**b**) and Abca3 (**c**) were downregulated. Mechanical ventilation did not cause further changes in gene expression. The quantification of RNA expression was based on the 2^△△CT^- method and HPRT was used as the housekeeper gene. Western Blots and densitometry measurements were used to quantify protein expression of proSP-B (**d**) and the 16 kDa (**e**) as well as 21 kDa (**f**) variant of proSP-C in the homogenate of lavaged lungs. In (**g**) representative Western Blots of proSP-B are shown while **h** illustrates representative Western Blots of proSP-C. Statistical analyses are based on a two-way ANOVA with the factors “bleomycin pre-treatment” and “mechanical ventilation”. Statistical significant differences after adjustment of the *p* level for multiple testing using Tukey correction is indicated as follows:*p < 0.05; *ns* not significant

After having characterized the intracellular surfactant pool based on ultrastructural criteria, we investigated the expression of important components of the intracellular surfactant system. The data are illustrated in Fig. 7. At the RNA level, we made use of quantitative real-time PCR to gain information on the RNA expression of the surfactants proteins B and C as well as Abca3 (Fig. 7a–c). For Abca3 and SP-C, the two-way ANOVA showed a significant downregulation of expression after bleomycin treatment at the RNA level (*p* < 0.0001). Direct comparison of groups H/no_ventil and B/no_ventil, demonstrated a significant difference on RNA level for Abca3 (*p* = 0.0064) and SP-C (*p* = 0.0066). Considering SP-B, no relevant downregulation could be observed at the RNA level. In order to complement the expression analyses of surfactant proteins B and C at the protein level Western Blots were performed and bands were quantified by means of densitometry. For protein-level investigation, proSP-B and proSP-C are the variants which are usually found in AE2 cells, including the LB, so that lung homogenates were generated and used for Western Blots (Fig. 7d–h). Two independent blots for proSP-B and three independent blots for proSP-C were carried out and quantified. Each blot contained 2 samples per study group which were averaged. Examples are illustrated in Fig. 7g, h. For proSP-C at 16 kD, we were able to demonstrate a significant downregulation of its production due to bleomycin challenge (*p* = 0.0048). For proSP-C 21 kD and proSP-B, however, no relevant downregulation could be found. Because no ventilation effects were observed at the RNA level we did not expect to find ventilation effects at the protein level and, as such, the statistical analyses focused on the bleomycin effects independent of ventilation. To conclude, these results indicate a significant effect of bleomycin treatment on lung surfactant metabolism, characterized by downregulation of the production of SP-C and Abca3 within lung parenchyma after intratracheal Bleomycin at the RNA level and, in part, also at the protein level. Mechanical ventilation did not have a clear effect on the expression levels of surfactant proteins.

### Correlation analyses

**Fig. 8:**
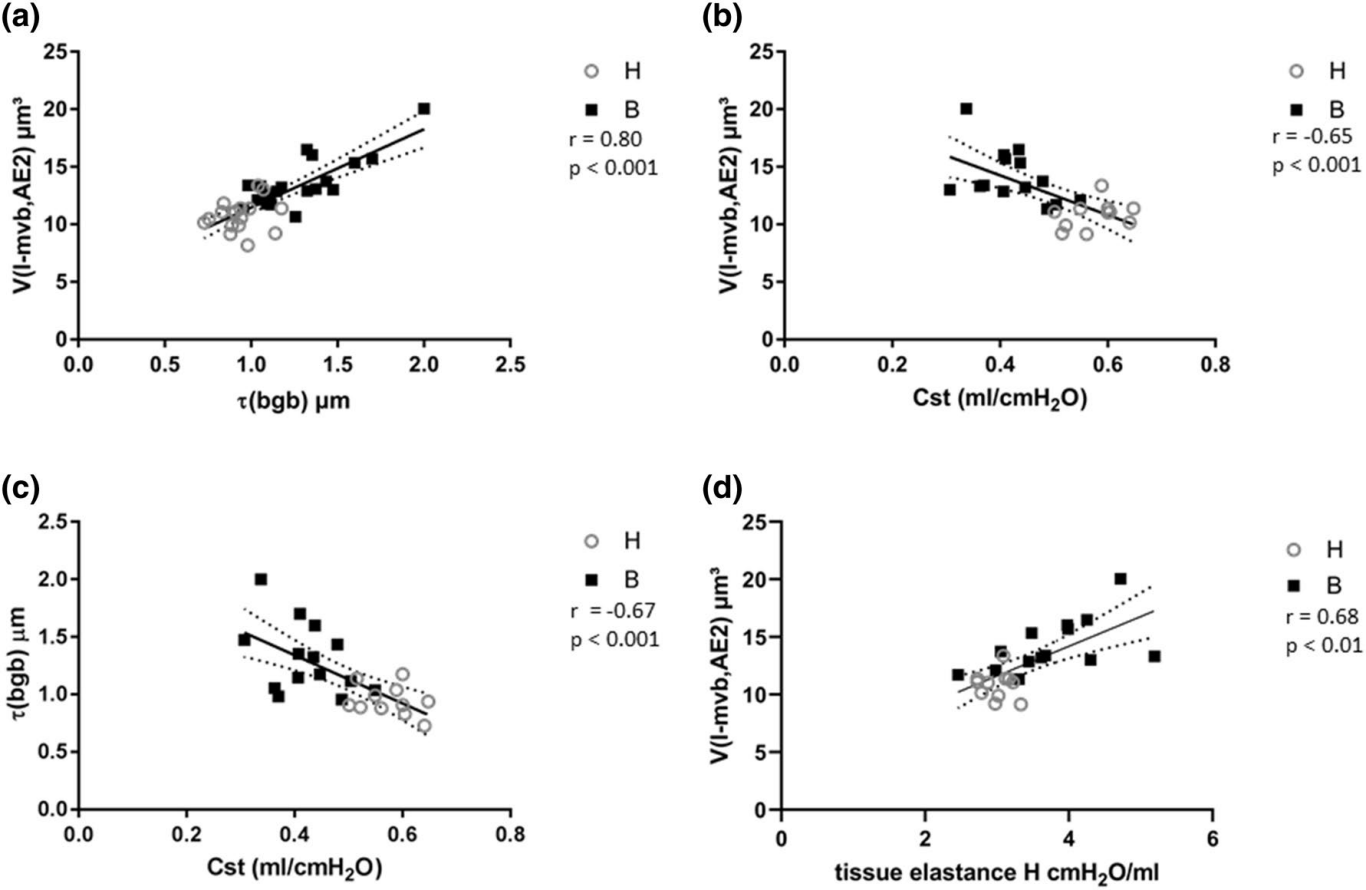
Correlation between structural and lung mechanical data. The volume of electron-lucent multivesicular bodies (l-mvb) per AE2 cell demonstrates a positive correlation with the arithmetic mean thickness of the blood–gas barrier (**a**) and a negative correlation with the quasi-static compliance (**b**). The arithmetic mean thickness of the blood–gas barrier correlated negatively with the quasi-static compliance (**c**). Tissue elastance H, based on impedance data from forced oscillation perturbations at the conclusion of the mechanical ventilation demonstrated a positive correlation with the volume of electron-lucent multivesicular bodies (**d**). The tissue elastance data shown in **d** were taken from a previous publication (Albert et al. 2020). The Pearson correlation coefficient r and statistical significant *p* are given. Linear regression was used to fit a line to the measured data. The 95% confidence interval of the fitted line is given by the dashed borders

Using the Pearson correlation coefficient, we characterized the relationship between the alterations of the ultrastructure of the AE2 cells, lung mechanical properties and ultrastructural changes of the blood–gas barrier in our experimental groups. Parameters related to the intracellular surfactant pool, such as the number and size of LB, did not correlate with markers of interstitial abnormalities ($\tau$(bgb)) or the lung mechanical properties (Cst). However, the volume of l-mvb per AE2 cell demonstrated a good correlation with both the thickness of the blood–gas barrier $\tau$(bgb) (Fig. 8a) and an inverse correlation with the Cst (Fig. 8b). Also, the thickness of the blood–gas barrier correlated inversely with Cst (Fig. 8c). In our previous study we characterized the mechanical properties during mechanical ventilation and repetitively measured the tissue elastance by means of the forced oscillation perturbation (Albert et al. 2020). The final measurements of tissue elastance, which was carried out during PEEP = 3 cmH_2_O ventilation were taken from that study for correlation with ultrastructural properties of AE2 cells. Here, we could confirm a strong correlation between *V*(l-mvb,AE2) and lung mechanics based on tissue elastance (Fig. 8d).

## Discussion

We investigated the synergistic effects of early bleomycin-induced lung injury and volume-controlled mechanical ventilation with different PEEP levels on the ultrastructure of the blood–gas barrier and on AE2 cells with a focus on the intracellular surfactant pool. Bleomycin-induced lung injury without mechanical ventilation results in larger but fewer LB in AE2 cells compared to healthy controls without mechanical ventilation (Fig. 5c, 5d). Similar abnormities of the ultrastructure of the intracellular surfactant pool have been reported in other animal models of acute lung injury (Knudsen et al. 2011; Fehrenbach et al. 1998a). In human donor lungs, such alterations were linked with the occurrence of primary graft dysfunction (which manifests as an acute lung injury) of the contralateral, transplanted lung (Fehrenbach et al. 1998b). In the present study, these structural observation were linked with a decreased expression of SP-C (Fig. 7b, e), an increase in the volume of electron-lucent multivesicular bodies (l-mvb) but a decrease in the volume of electron-dense multivesicular bodies (d-mvb) per AE2 cells after bleomycin challenge without mechanical ventilation (Fig. 6). Taking the volumes of l-mvb and d-mvb per AE2 cell together, however, no differences could be found between bleomycin-challenged and healthy non-ventilated lungs (Table 3).

Previous studies have demonstrated a lack of intra-alveolar surfactant and a significant reduction in the surface tension reducing properties of broncho-alveolar lavage-derived surfactant one day after bleomycin challenge (Lutz et al. 2015). Furthermore, there was a decrease in tubular myelin and lamellar body-like structures within the alveolar space (Lutz et al. 2015), which may indicate that the bleomycin challenge results in a decrease in secretion of LB into the alveolar space. In the present study, we observed that some components relevant for the biosynthesis of functional surfactant were down-regulated after bleomycin-induced lung injury and this included SP-C at the RNA and protein levels (Fig. 7b, e). ProSP-C levels measured in the homogenate of lavaged lungs show a clear bleomycin-induced reduction of 16 kDa proSP-C; while, the 21 kDa proSP-C appeared to be less affected. The intracellular processing of proSP-C is complex and includes appropriate folding, post-translational palmitoylation as well as a number of cleavage events so that several cellular compartments such as the rough endoplasmic reticulum, the Golgi apparatus, the mvb and the LB are involved (Beers and Mulugeta 2005). Using immunogold labeling and design-based stereology, proSP-C has been preferentially found in mvb (Schmiedl et al. 2005). In cell culture experiments, the 21 kDa proSP-C could be isolated predominantly from microsomes (usually in the rough endoplasmic reticulum and cellular vesicles); while, the 16 kDa proSP-C was predominantly observed in LB. Therefore, the 16 kDa proSP-C was considered to be an intermediate variant within the processing pathway to mature 3.6 kDa SP-C which is localized in LB within the cell (Beers et al. 1994). The present study indicates a link between ultrastructural abnormalities of LB and a reduction in levels of 16 kDa proSP-C in lung homogenate after bleomycin challenge, suggesting that bleomycin injury interferes with the necessary transformation of 21–16 kDa proSP-C.

Previous studies have already shown that the surfactant proteins are downregulated after bleomycin challenge during the acute and fibroproliferative phase of that injury model (Schmidt et al. 2004). In the current study, we presented the first observations demonstrating disturbances of intracellular surfactant including down-regulation of SP-C at a very early time point and before clinical manifestation of the lung injury. The effects of bleomycin were also manifested in the blood–gas barrier that showed a slight increase in thickness when comparing pre-injured and healthy lungs prior to mechanical ventilation (Fig. 2c). Abnormalities in intracellular surfactant homeostasis were already present at the early 1-day time point at which injury-related alterations in the interstitial space of the interalveolar septa were quite mild and hard to detect. Widening of the interstitial layer within the interalveolar septa is a typical feature of acute lung injury in animal models (Matute-Bello et al. 2011; Mühlfeld et al. 2009) but has also been demonstrated at electron microscopic level in patients suffering from acute respiratory distress syndrome (Bachofen and Weibel 1977).

Aside from larger and fewer LB per AE2 cells in non-ventilated pre-injured lungs compared to non-ventilated healthy lungs, the volume per AE2 cell of l-mvb was increased, while the volume of d-mvb was decreased in none-ventilated pre-injured lungs (Fig. 6). Multivesicular bodies have been suggested to be involved in lipid sorting after re-uptake of used surfactant from the alveolar space, e.g., by shifting surfactant material directly into the LB (recycling) or to the lysosomal compartment for degradation and re-syntheses. During the de novo biogenesis of LBs, the mvb have been discussed to be involved in the transport of surfactant components from the Golgi apparatus to the LB (Chevalier and Collet 1972; Rooney et al. 1994; Wright and Dobbs 1991; Rider et al. 2000). In other words, there is some evidence that mvb link the endosomal and the secretory cellular compartment in AE2 cells (Williams 1984; Kalina and Socher 1990). Used alveolar surfactant, also referred to uni-lamellated vesicles, is endocytosed and might be shifted into l-mvb which are free of lysosomal enzymes and might directly recycle the surfactant material to the LB, a process which has been suggested to be dependent on lectins such as surfactant protein A (Williams 1984; Bates et al. 2008; Cañadas et al. 2020). Otherwise, surfactant material might also be sorted, e.g., in the absence of sufficient SP-A, to the degradation pathway and this pathway has been suggested to involve the d-mvb which contain lysosomal enzymes and no SP-A (Williams 1984; Rooney et al. 1994; Young et al. 1993). In the present study, we observed a slight increased volume of l-mvb 1 day after bleomycin challenge in non-ventilated pre-injured lungs and this might reflect increased endocytosis of inactivated surfactant material from the alveolar space. However, the pathways involved in surfactant homeostasis are highly dynamic and in our present study we analyzed components involved within this dynamic process under static conditions. From these data, it is not possible to deduce the functional state of l-mvb and, therefore, the rate of recycling. Using healthy lungs, Young and co-workers investigated the time course and involved cellular organelles of surfactant uptake into AE2 cells by means of autoradiography of labeled phospholipids (Young et al. 1993). Based on these observations, the authors speculate that the turnover rates of surfactant lipids in the l-mvb seem to be very rapid and much higher than the turnover rates in LB. Hence, increased intracellular volumes of l-mvb might reflect increased but ineffective recycling due to a delayed shift of surfactant material into the LB or the degradation pathway. Although mvb are relatively frequently observed in AE2 cells (Mulugeta et al. 2015), they are also found in other cell types (Rooney et al. 1994) and they serve a diversity of cellular functions, so that the observed alterations might also be a consequence of cellular reactions independent of the surfactant metabolism. Nevertheless, it can be concluded from the data of the present study that the bleomycin-pre-injured lungs start into the mechanical ventilation with an altered intracellular surfactant system at a time point where signs of acute lung injury such as interstitial oedema and increased thickness of the blood–gas barrier are subtle.

In healthy lungs, volumes of both l-mvb and d-mvb per AE2 cell remained very stable across all groups so that there is no hint at an increased recycling or de novo synthesis based on these structural data. There was a slight down-regulation at the RNA level of SP-C and Abca3 in healthy lungs ventilated with PEEP = 5 cmH_2_O compared to non-ventilated heathy lungs (Fig. 7b and 7c). Nevertheless, our structural data related to the mvb do not allow drawing conclusions on the functional state of these organelles.

The effects of mechanical ventilation on AE2 cells, intracellular surfactant pool and composition of the blood–gas barrier were different in healthy lungs compared to bleomycin-pre-injured lungs. In healthy lungs, the number-weighted mean volume of AE2 cells decreased with increasing PEEP (Fig. 2a) and this was accompanied by a decrease in the total volume of LB per cell (Fig. 2b). In addition, the number of LB per AE2 cell was stable during mechanical ventilation with increasing PEEP level in the healthy lungs (Fig. 5c) so that the number-weighted mean volume of LB trended towards a decrease (Fig. 5d). It is of utmost importance that the intra-alveolar surfactant pool size is tightly regulated to guarantee a sufficient quantity of functional surfactant in the airspace. Alveolar stretch has been shown to be an important stimulus for exocytosis of LB in vitro (Wirtz and Dobbs 1990). Further studies have shown that deep inflations as well as hyperventilation increased the amount of phospholipids in the broncho-alveolar lavage fluid and this is most likely due to a shift of surfactant from the intracellular into the intraalveolar surfactant pool by exocytosis of LB (Wright and Dobbs 1991). Based on the ultrastructural investigations, deep inflations, corresponding to a volume of four times the tidal volume, which were performed every 5 min over a ventilation period of 60 min led to a decrease of the volume fraction of LB within AE2 cells by one third, an observation which provided evidence for deep inflation-induced surfactant secretion (Massaro and Massaro 1983). Milos et al*.* investigated the intracellular surfactant pool after high tidal volume ventilation (25–30 ml/kg bodyweight), PEEP = 0 cmH_2_O and 100% inspiratory oxygen content (F_IO2_ = 1) in primarily healthy lungs (Milos et al. 2017). These lungs developed VILI and demonstrated a decrease in the area of AE2 cells occupied by LB, a parameter that corresponds to the volume fraction of LB within AE2 cells. In the present study, the volume fraction of LB within AE2 cells did not differ between ventilated and non-ventilated healthy lungs (Table 3), so that we could not reproduce these previous observations (Milos et al. 2017; Massaro and Massaro 1983) in terms of the volume fractions. This is most likely due to a different ventilation protocol using room air, PEEP > 0 cmH_2_O, a tidal volume of 10 ml/kg bodyweight and deep inflations only once per hour in the present study. The airway opening pressure during a deep inflation was limited to 30 cmH_2_O so that the lung volume at the end of the deep inflation corresponded approximately to the total lung capacity. Nevertheless, with increasing PEEP level during mechanical ventilation, we observed a decrease in the absolute volume of LB per AE2 cell along with a reduced cell volume of AE2 cells so that the volume fraction of LB within the AE2 cells remained stable at the end of ventilation. In the context of the mentioned literature, the decrease in the volume of LB per AE2 cell can be interpreted in a way that with increasing PEEP during mechanical, there is a shift of surfactant material from the intracellular surfactant pool into the alveolar space.

While in healthy lungs the number of LB remains stable (Fig. 5c), the number-weighted mean volume of LB decreases (Fig. 5d) and the coefficient of variation of LB size increase with increasing PEEP. These data suggest that in this dynamic process, larger LB are secreted and smaller LB are formed during mechanical ventilation (Chevalier and Collet 1972). The effect of mechanical ventilation on the intracellular surfactant pool was much more pronounced after PEEP = 5 cmH_2_O than after PEEP = 1 cmH_2_O ventilation in healthy lungs. Mechanical ventilation induces deformation not only at the organ scale but also at the micromechanical, alveolar scale and these deformations can be separated into a static and dynamic component (Knudsen and Ochs 2018; Güldner et al. 2016). Increasing PEEP results in a static deformation or strain, while the tidal volume imposes dynamic alveolar strain (Knudsen et al. 2018). In the present study, the ventilation-induced alterations of the intracellular surfactant pool increase with the static alveolar strain.

The behavior of the intracellular surfactant pool after mechanical ventilation was different in the bleomycin-challenged lungs compared to healthy lungs. With bleomycin, the total volume of LB per AE2 cell remained stable even with increasing PEEP levels, i.e., increasing static strain, so that there is a lack of evidence for a stretch induced net shift of surfactant material from the intracellular to the intraalveolar pool. In contrast, healthy lungs showed a pronounced PEEP-dependent decrease in number-weighted mean volume of lamellar bodies. This behavior was linked to a dramatic increase in the coefficient of variation of LB sizes. In the bleomycin case, there are more LB with a higher size variability, and stable absolute LB volume per AE2 cell across groups. This finding can be explained by formation of new, smaller LB that replace larger LB in a way that the absolute intracellular surfactant volume remains roughly stable. In addition, the bleomycin-pre-injured lungs ventilated with a PEEP of 1 cm H_2_O, but not with a PEEP of 5 cmH_2_O, show a significant increase in the volumes of l-mvb and d-mvb per AE2 cell (Fig. 6), providing further evidence of structural alterations that differentiate the response of AE2 cells in pre-injured compared to healthy lungs.

Although interstitial abnormalities within the interalveolar septa were quite subtle at this very early time point after bleomycin challenge, the different PEEP levels chosen during mechanical ventilation had different effects on the ultrastructural composition of the blood–gas barrier. After mechanical ventilation of bleomycin-pre-injured lungs with PEEP = 5 cmH_2_O, the interstitial abnormalities were significantly reduced compared to mechanical ventilation with PEEP = 1 cmH_2_O. In other words, PEEP = 1 cmH_2_O appeared to aggravate interstitial abnormalities while PEEP = 5 cmH_2_O protected against structural degradation, leading to significant greater septal interstitial tissue volume and elevated arithmetic mean thickness of the blood–gas barrier in pre-injured lungs ventilated with PEEP = 1 cmH_2_O (Fig. 2c, d). Hence, these data provide evidence that low PEEP ventilation aggravates lung injury in at-risk lungs, such as the bleomycin-pre-injured lung. In contrast, the healthy lungs did not demonstrate these ultrastructural changes when ventilated at low PEEP and a tidal volume of 10 ml/kg. These observations support recent findings in mechanically ventilated, bleomycin-injured lungs showing a progressive increase in tissue elastance measured with the forced oscillation technique during mechanical ventilation with low but not with high PEEP, a behavior which highly correlated with BAL protein and albumin levels (Albert et al. 2020).

It is well established that mechanical ventilation of initially healthy lungs becomes harmful when low PEEPs are combined with high tidal volumes (Seah et al. 2011; Szabari et al. 2019). When lung injury is induced with bleomycin 1 day prior to ventilation, low PEEP ventilation is linked with microatelectases and surfactant insufficiency (Knudsen et al. 2018). These microatelectases are likely to act as stress concentrators (Mead et al. 1970; Makiyama et al. 2014) and increase the dynamic alveolar strain in otherwise healthy alveoli that are in close proximity to collapsed alveoli (Albert et al. 2019; Knudsen et al. 2018). These mechanisms can explain why the PEEP = 1 cmH_2_O, unlike PEEP = 5 cmH_2_O, aggravated interstitial abnormalities in bleomycin-pre-injured lungs. However, we could not establish a convincing relationship between abnormalities in the intra-cellular surfactant pool and the interstitial abnormalities found after bleomycin challenge and mechanical ventilation with different PEEP levels. It seems that the ventilation-induced abnormalities related to the intracellular surfactant pool are not linked to the changes of the septal interstitium. On the contrary, the absolute volumes of l-mvb per AE2 cell increased after bleomycin challenge with PEEP = 1 cmH_2_O but not with PEEP = 5 cmH_2_O so that this parameter behaved similar to the thickness of the blood–gas barrier. Accordingly, a strong positive correlation between the volume of l-mvb per AE2 cells and the arithmetic mean thickness of the blood–gas barrier was identified (Fig. 8a), indicating that these two parameters are influenced by the same factors or are directly dependent on each other, e.g., due to a causal relationship. Since microatelectases exist when ventilating the bleomycin-injured lungs at PEEP = 1 cmH_2_O, which results in an increase in mean intra-tidal alveolar strain at the micromechanical level (Knudsen et al. 2018), we postulate that both the increased volume of l-mvb per AE2 cell and the increased arithmetic mean thickness of the blood–gas barrier are stretch induced. However, the pathophysiological role of mvb in the context of lung injury requires further investigations. In addition to quantitative morphological data, we also measured the quasi-static compliance (Cst) based on PV loops at the end of the mechanical ventilation. The bleomycin-pre-injured lungs had a significantly decreased Cst and this decrease in Cst was very convincingly linked to the interstitial abnormalities, e.g., an increase of the arithmetic mean thickness of the blood–gas barrier (Fig. 8c) but also the volume of l-mvb per AE2 cell (Fig. 8b). Therefore, these two structural parameters reflect abnormalities in the lung mechanical properties.

The bleomycin animal model mimics some relevant features of acute lung injury (Matute-Bello et al. 2011) and has therefore been used quite frequently in the past (Matute-Bello et al. 2008). In the context of alveolar micromechanics, there is some evidence that bleomycin introduces similar abnormalities in terms of the distribution of opening pressures of distal airspaces compared to human ARDS (Knudsen et al. 2018; Cressoni et al. 2017). The described abnormalities in the ultrastructure of the intracellular surfactant induced by bleomycin are partly shared with other models of acute lung injury such as the endotoxin or ischemia/ reperfusion-induced lung injury (Fehrenbach et al. 1998a) and might also be of relevance in human lungs regarding the development of manifest acute lung injury after lung transplantation (Fehrenbach et al. 1998b). Nevertheless, it remains unclear whether or not the described effects of mechanical ventilation on the ultrastructure of AE2 cells in the present study can be generalized to other animal models of lung injury or even the human lung.

In summary, we illustrated that bleomycin challenge of the lung resulted in ultrastructural abnormalities of the AE2 cells that preceded the clinical manifestation of the acute respiratory distress syndrome-like phase of the model. Mechanical ventilation with PEEP = 1 cmH_2_O, but not with PEEP = 5 cmH_2_O, aggravated interstitial abnormalities within the interalveolar septa resulting in a thickening of the blood–gas barrier. Invasive mechanical ventilation resulted in different ultrastructural alterations of the intracellular surfactant pool and the volumes of mvb per AE2 cell in bleomycin-pre-injured lungs compared to healthy lungs. While alterations of the intracellular surfactant were not linked to mechanical ventilation-induced aggravation of interstitial abnormalities or lung mechanical properties, the volumes of mvb per AE2 cell demonstrated a high correlation with both quasi-static compliance of the respiratory system and the thickness of the blood–gas barrier. However, the impact of mechanical ventilation on the functional state of the mvb, e.g., in the context of surfactant metabolism, as well as pathophysiological consequences in the context of VILI, requires further investigations.
